# Effect of Soleus Muscle Shear Modulus on Deep Squatting Motion at Increased Ankle Dorsiflexion

**DOI:** 10.7759/cureus.87746

**Published:** 2025-07-11

**Authors:** Yusuke Murofushi, Nana Hirata, Katsuhiko Suzuki

**Affiliations:** 1 Department of Physical Therapy, Yamagata Prefectural University of Health Sciences, Yamagata, JPN

**Keywords:** ankle dorsiflexion, deep squat, muscle stiffness, shear modulus, soleus muscle

## Abstract

Introduction

Deep squats are important functional movements in many Asian cultures, and the ability to perform this movement is a key indicator of lower-limb function. Ankle joint range of motion, particularly dorsiflexion, plays an important role in deep squats. This study aimed to investigate the relationship between muscle stiffness in the triceps surae (medial and lateral heads of the gastrocnemius and the soleus) and the ability to perform deep squatting motions, as measured by the shear modulus.

Methods

A total of 45 healthy participants were recruited for this study, including 24 males (53.3%) and 21 females (46.7%). We measured the stiffness (shear modulus) of the gastrocnemius and soleus muscles at various ankle dorsiflexion angles (0-40°) in the flexed knee position. The participants were divided into a "possible group" (those able to perform a deep squat with heels on the floor) and an "impossible group" (those unable to perform the deep squat with heels on the floor). The shear modulus values of the medial and lateral heads of the gastrocnemius and the soleus muscle were compared between the two groups.

Results

The shear modulus of the soleus muscle at 40° of ankle dorsiflexion was significantly lower in the impossible group (14.09 ± 6.79 kPa) than in the possible group (9.37 ± 5.55 kPa, p < 0.05). The intraclass correlation coefficient (ICC) values for the muscle measurements ranged from 0.75 to 0.89, indicating good reproducibility.

Conclusion

This study showed that soleus muscle stiffness at 40° ankle dorsiflexion differed between individuals who can and cannot perform deep squats. This suggests that insufficient elongation of the soleus muscle may hinder the achievement of the dorsiflexion angle necessary for deep squats.

## Introduction

The deep squatting motion is an important lifestyle movement in many Asian countries, including Japan, and has been reported to influence quality of life (QOL) [1]. Additionally, deep squatting is a key indicator for evaluating lower limb function and may be restricted by certain diseases [2-6]. Therefore, reacquiring the ability to deep squat is believed to contribute to improving QOL and promoting return to work. The range of motion (ROM) of the ankle joint significantly affects deep squatting motion [7]. For example, a greater range of ankle dorsiflexion in a flexed knee position allows for a deeper squat [8], indicating that ankle flexibility plays a critical role in enabling proper deep squatting posture [9].

Flexibility is typically assessed using indicators such as joint ROM and joint stiffness. Joint stiffness is calculated by dividing joint torque by the change in joint angle and is influenced by muscle stiffness [10]. Muscle stiffness can be estimated non-invasively using ultrasound shear wave elastography (SWE), a technique that measures the speed of shear waves propagating through tissue; faster wave speeds indicate stiffer tissues [11]. The shear modulus, derived from shear wave speed and tissue density, reflects muscle stiffness and correlates with Young’s modulus in material testing; a higher shear modulus suggests the muscle is in a stretched state [12]. The shear modulus of the triceps surae muscles affects ankle dorsiflexion ROM [13] and is known to vary by sex and age [10,14]. Its measurement has shown good reproducibility [13,15]. However, most previous studies have assessed shear modulus in an extended knee position, while deep squatting with heels on the ground typically involves an average dorsiflexion angle of 35.8° at the ankle when the knee is flexed [16]. In the flexed knee position, the gastrocnemius is relaxed, suggesting that the flexibility of the soleus muscle (SOL) becomes more influential in deep squatting [17].

Therefore, this study aimed to investigate the relationship between the shear modulus of the triceps surae and deep squatting motion when the dorsiflexion angle of the ankle joint is changed from 0° to 40° in a flexed knee position and to clarify the level of muscle flexibility required to achieve deep squatting.

## Materials and methods

Participants

This study included 45 participants, comprising 24 healthy males (53.3%) and 21 females (46.7%), with an average height of 164.3 ± 8.0 cm, an age of 20.7 ± 1.2 years, and a weight of 57.8 ± 7.9 kg. Participants were instructed to refrain from vigorous exercise for 24 hours prior to the measurement day. None of the participants reported muscle pain or neurological disorders during the experiment. Additionally, only individuals with no history of lower limb disease were included. All participants were provided with both verbal and written explanations of the study, and only those who provided written consent were included. This study was approved by the Ethics Committee of Yamagata Prefectural University of Health Sciences (2308-16).

Positioning

**Figure 1:**
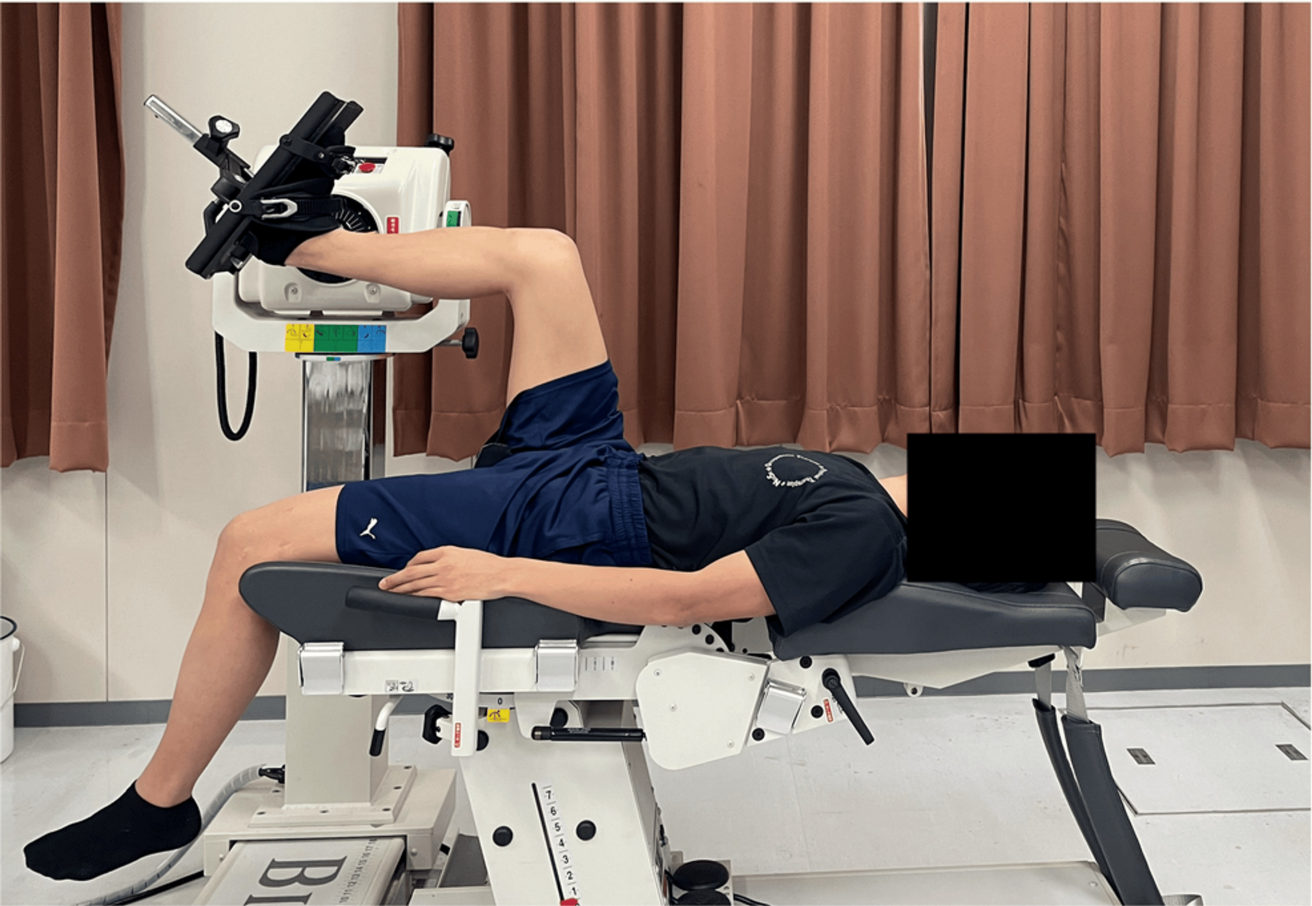
Measurement posture in the supine position with the hip and knee joints flexed to 90° The posture adopted during the measurements is illustrated. Shear wave velocity was measured as the ankle dorsiflexion angle was varied from 0° to 40°.

The participants were positioned supine on a Biodex isokinetic dynamometer (Biodex Medical Systems; Biodex System 4, California, USA) with the knee and hip joints flexed at 90°, their feet fixed on a footplate, and the ankle in a neutral position. To familiarize the participants with the passive dorsiflexion exercise using Biodex, the ankle was passively moved prior to measurement. Changes in ankle angle were measured from 0° plantar flexion to 40° dorsiflexion, with measurements taken every 10°. If pain occurred or it was difficult to maintain the measurement position, the measurements were not conducted. Participants were verbally instructed to relax and minimize tension during measurements (Figure 1).

Shear wave elastography (SWE)

**Figure 2:**
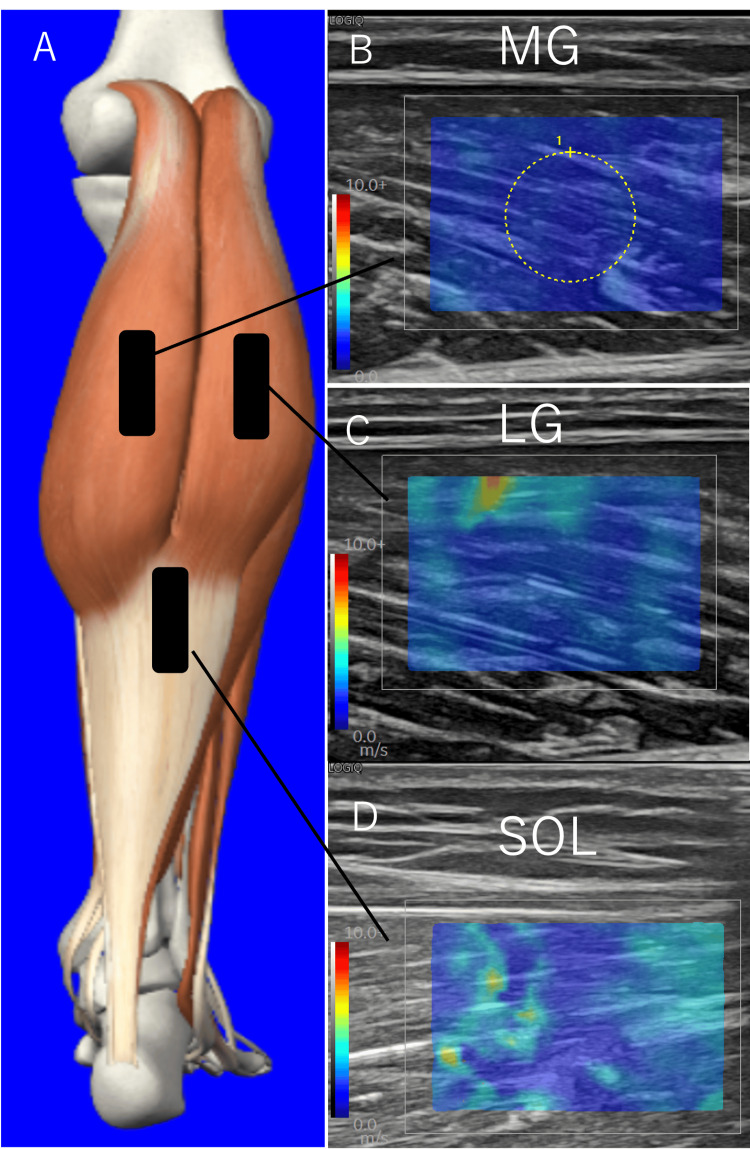
Schematic representation of ultrasound probe locations and typical examples of ultrasound shear wave elastographic images (A) Anatomical illustration of the triceps surae showing the probe placement sites (black rectangles) for elastography measurement. (B) Elastography image of the medial gastrocnemius (MG). (C) Elastography image of the lateral gastrocnemius (LG). (D) Elastography image of the soleus muscle (SOL). The rectangular boxes in panel A indicate the approximate locations where the ultrasound probe was placed to obtain the elastography images shown in panels B-D. A circular region of interest (ROI) with a diameter of 5 mm was used for the shear wave velocity measurements.

An ultrasound imaging system (GE Healthcare; LOGIQ P10, Chicago, USA) with a linear probe (GE Healthcare; L3-12-RS) was used to measure shear wave velocity (SWV). The target muscles were the medial head of the gastrocnemius (MG), the lateral head of the gastrocnemius (LG), and the SOL. The position of the probe was set at 30% of the lower leg length proximal to the MG and LG [14]. The probe was placed on the distal part of the SOL, between the distal muscle-tendon junctions of the MG and LG and the distal muscle-tendon junction of the soleus [18]. The probe was positioned along the direction of the fascicle fibers of the target muscle, using sufficient ultrasound gel to avoid excessive pressure. Subsequently, the angle of the probe was adjusted to align with the direction of the fascicle fibers of each muscle. The measurement was performed by setting the region of interest (ROI) at the boundary between the subcutaneous tissue and the muscle, with a circle of 5 mm diameter placed at the center of the ROI (Figure 2). Then, within the circle in the quantitative analysis, the SWV and Young’s modulus were automatically calculated. The measurements were performed three times, and the average SWV values were used for the analysis. In the present study, shear modulus (µ) was used as an index of tissue stiffness and calculated using the following formula: $\mu\ (\text{kPa}) = \rho \cdot V^2$, where V represents the SWV and ρ is the muscle mass density (1000 kg/m^3^) [12]. The measurements were conducted with the muscle relaxed as it elongated in response to changes in the ankle angle.

Grouping

**Figure 3:**
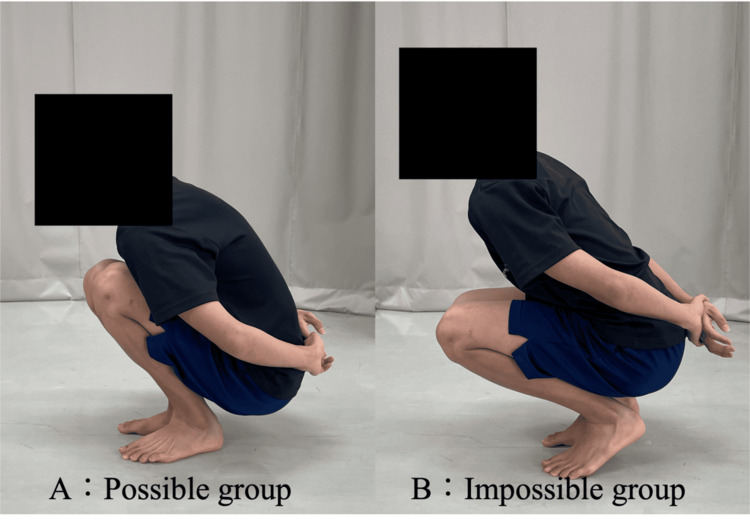
Grouping The criteria for classification were as follows: the “possible group” consisted of participants who could perform a deep squat with their hands behind their back while keeping their heels on the floor, whereas the “impossible group” consisted of participants who could only perform a squat by lifting their heels off the floor. Images A and B show the possible and impossible groups, respectively.

Participants who could perform a deep squat with their arms crossed behind their back while keeping both heels in contact with the floor and without leaning backward were classified as “possible group.” Conversely, those unable to maintain heel contact with the floor during a deep squat were classified as “impossible group.” On the frontal plane, the feet were positioned shoulder-width apart, and verbal instructions were provided to ensure that the knees did not turn outward during the movement. The ability to perform deep squats was confirmed at least twice, and the participants were classified into groups accordingly (Figure 3 and Table 1).

**Table 1:** Physical characteristics of each group

Characteristic	Possible group (n = 25)	Impossible group (n = 20)
Sex (men: women)	12:13	12:8
Height (cm)	162.0 ± 6.3	167.3 ± 8.8
Weight (kg)	55.8 ± 7.0	60.4 ± 8.2
Age (years)	20.6 ± 1.1	20.8 ± 1.3
BMI	21.5 ± 2.0	21.5 ± 1.6

Statistical analyses

The shear modulus of the MG, LG, and SOL at each joint angle was compared between the possible and impossible groups using a two-sample t-test. In addition, the reproducibility of the shear modulus was confirmed using the intraclass correlation coefficient (ICC). Statistical analyses were conducted using IBM SPSS Statistics for Windows, Version 25 (Released 2017; IBM Corp., Armonk, New York, United States), with a significance level of 5%. Effect size was measured using Cohen’s d. The reliability of the shear modulus measurements for each group and muscle was assessed using the ICC and coefficient of variation (CV).

## Results

The possible group comprised 25 participants (62.5%), whereas the impossible group comprised 15 participants (37.5%). In the impossible group, one participant (6.7%) could not perform ankle dorsiflexion at 15°, and four participants (26.7%) could not perform ankle dorsiflexion at 20°. Therefore, in the impossible group, data for ankle dorsiflexion at 15° included 14 participants (93.3%), and data for dorsiflexion at 20° included 11 participants (73.3%).

Table 2 lists the shear moduli of the three muscles at each dorsiflexion angle. For the SOL at 40° ankle dorsiflexion, the shear modulus was 14.09 ± 6.79 kPa in the possible group and 9.37 ± 5.55 kPa in the impossible group (p < 0.05, effect size = 0.77). Table 3 shows the ICC (ICC 1.1) and CV for each muscle. The ICC for the MG (possible group, impossible group) was 0.88 and 0.89, LG (0.89,0.88), and SOL (0.75, 0.76). The CV values for MG were (30.5, 21.9), LG (33.8, 28.0), and SOL (33.9, 31.2).

**Table 2:** Shear modulus (kPa) at different dorsiflexion angles *Significantly different from the impossible group (p < 0.05). In the impossible group, data on ankle dorsiflexion at 15° was included for 14 subjects, and data on dorsiflexion at 20° was included for 11 subjects. MG: medial heads of gastrocnemius; LG: lateral heads of gastrocnemius; SOL: soleus

Muscle	Group	0°	10°	20°	30°	40°
MG	Possible group	3.96 ± 3.30	4.79 ± 1.78	7.68 ± 3.65	10.79 ± 4.40	13.19 ± 5.71
	Impossible group	4.37 ± 1.44	5.80 ± 2.40	8.15 ± 3.02	12.20 ± 4.33	16.23 ± 6.23
LG	Possible group	3.36 ± 1.00	3.72 ± 1.33	4.42 ± 1.54	5.93 ± 3.45	7.82 ± 4.42
	Impossible group	3.53 ± 0.74	4.03 ± 1.69	5.07 ± 2.88	7.03 ± 5.06	8.39 ± 5.81
SOL	Possible group	4.73 ± 2.25	4.75 ± 2.12	7.78 ± 4.39	9.42 ± 5.53	14.09 ± 6.79*
	Impossible group	4.53 ± 2.40	5.41 ± 2.97	8.64 ± 4.07	12.01 ± 8.94	9.37 ± 5.55

**Table 3:** Values of ICC and CV for each muscle MG: medial heads of gastrocnemius; LG: lateral heads of gastrocnemius; SOL: soleus; ICC: intraclass correlation coefficients; 95％ CI: 95％ confidence interval; CV: coefficient of variation

Muscle	Group	ICC (1,1)	95% CI	CV (%)
MG	Possible group	0.89	0.86-0.92	30.5
	Impossible group	0.88	0.84-0.91	21.9
LG	Possible group	0.89	0.86-0.92	33.8
	Impossible group	0.88	0.84-0.92	28.0
SOL	Possible group	0.75	0.68-0.81	33.9
	Impossible group	0.76	0.70-0.83	31.2

## Discussion

In this study, we used shear modulus to examine whether there were differences in muscle stiffness among the MG, LG, and SOL depending on the ability to perform deep squats. As a result, the shear modulus of SOL at 40° ankle dorsiflexion was lower in the group that could not perform deep squats than in the group that could. It has been shown that muscle stiffness is correlated with muscle elongation, which indicates that muscles are stretched [12]. Therefore, the results of this study suggest that individuals who had difficulty performing the squatting motion did not experience elongation of the SOL at 40° ankle dorsiflexion.

It has been reported that the elasticity of the Achilles tendon increases when the ankle joint moves into dorsiflexion [19], and the shear wave speed of the Achilles tendon has been shown to influence dorsiflexion movements. The Achilles tendon is composed of the MG, LG, and SOL. During passive ankle dorsiflexion, the superficial portion of the Achilles tendon elongates more than the deeper portion [20]. The Achilles tendon consists of a superficial part formed by the gastrocnemius and a deep part formed by the soleus [21]; therefore, the SOL is considered to have a significant effect on dorsiflexion. Furthermore, during passive dorsiflexion in the flexed position, the gastrocnemius, which is a biarticular muscle, relaxes. Thus, the soleus likely plays a more significant role in influencing the stiffness of the Achilles tendon. The ankle dorsiflexion angle required for a deep squat was 35.4°, and because the SOL was not stretched to 40°, a sufficient dorsiflexion angle was not achieved, making it difficult to perform the squatting motion. The ICC values for the muscles measured in this study ranged from 0.75 to 0.89. Koo and Li [22] reported that an ICC between 0.75 and 0.90 indicates good reproducibility, suggesting that the reproducibility in this study was good. However, some studies reported ICC values > 0.90 for the triceps surae [10,15]. These studies primarily measured the muscles in the knee extension position, which differs from the measurement position in this study. Moreover, when the knee is flexed, the gastrocnemius relaxes and the measurement site becomes deeper. The lower reproducibility observed compared to previous studies is likely due to the increased measurement depth [23]. The CV ranged from 21.9% to 33.9%. Nakamura et al. reported a CV of 4.6-6.1% for the MG; therefore, the CV in the present study was higher. The variation in SWE values was also affected by the machine used, which likely contributed to the increased CV [15,24].

This study has the following limitations. First, the muscle contraction state was not monitored using electromyography during the experiment; therefore, the potential effects of muscle contraction on SWV were not fully considered. Second, the participants were limited to a young population, making it difficult to generalize the findings of this study. Third, muscle activity, including that of the tibialis anterior, which may influence deep squatting performance, was not considered. Further studies that take into account age and other relevant factors are necessary.

## Conclusions

In conclusion, this study demonstrated that the shear modulus of the SOL at 40° ankle dorsiflexion was lower in individuals who were unable to perform the squatting motion than in those who could. This suggests that insufficient elongation of the SOL during dorsiflexion may contribute to difficulty in achieving the necessary ankle dorsiflexion angle for squatting.
